# Altered Cardiac Autonomic Regulation in Individuals with Myasthenia Gravis—A Systematic Review and Meta-Analysis

**DOI:** 10.3390/neurolint15030071

**Published:** 2023-09-08

**Authors:** Monika Zawadka-Kunikowska, Łukasz Rzepiński, Małgorzata Tafil-Klawe, Nicola Veronese, Mario Barbagallo, Mario Habek, Nils E. Gilhus

**Affiliations:** 1 Department of Human Physiology, Nicolaus Copernicus University Ludwik Rydygier Collegium Medicum in Bydgoszcz, Karłowicza 24, 85-092 Bydgoszcz, Poland; malg@cm.umk.pl; 2 Sanitas—Neurology Outpatient Clinic, Dworcowa 110, 85-010 Bydgoszcz, Poland; luk.rzepinski@gmail.com; 3 Department of Neurology, 10th Military Research Hospital and Polyclinic, 85-681 Bydgoszcz, Poland; 4 Geriatrics Section, Department of Internal Medicine, University of Palermo, 90133 Palermo, Italy; nicola.veronese@unipa.it (N.V.); mario.barbagallo@unipa.it (M.B.); 5 Department of Neurology, Referral Center for Autonomic Nervous System Disorders, University Hospital Center Zagreb, 10000 Zagreb, Croatia; mhabek@mef.hr; 6 School of Medicine, University of Zagreb, 10000 Zagreb, Croatia; 7 Department of Neurology, Haukeland University Hospital, 5021 Bergen, Norway; nils.gilhus@uib.no; 8 Department of Clinical Medicine, University of Bergen, 5020 Bergen, Norway

**Keywords:** myasthenia gravis, cardiac, autonomic dysfunction, sympathovagal balance, heart rate variability

## Abstract

The aim of this systematic review with meta-analysis was to determine differences in cardiovascular autonomic parameters between patients with myasthenia gravis (MG) and healthy controls (HCs). Two reviewers searched four electronic databases, namely PubMed, Web of Science, EMBASE, and SCOPUS, from database inception to 7 July 2023 for studies investigating cardiovascular autonomic parameters in MG vs. HCs. A random-effects meta-analysis was performed to compute Hedges’ g ± 95% confidence intervals (CI). Out of a total of 2200 records, 8 observational studies with a sample size of 301 patients with MG and 454 HCs were included in the systematic review. Meta-analysis revealed lower values of expiration/inspiration ratio (g = −0.45, I^2^ = 74.7), baroreflex sensitivity (g = −0.56, 95%CI −0.80, −0.33; I^2^ = 0.3), percentage of adjacent NN intervals differing by more than 50 ms (g = −1.2, I^2^ = 82.8), square root of the mean of squared differences between successive beat intervals (g = −1.94, I^2^ = 95.1), mean of the standard deviations of all NN intervals (g = −0.83, 95%CI −1.37, −0.28; I^2^ = 55.5), and high frequency of HRV during tilt (g = −0.75, 95%CI −0.11, −0.39; I^2^ = 0). MG patients vs. HCs had higher systolic blood pressure (g = 0.39; I^2^ = 56.1), sympathovagal balance at rest/during tilt (LF/HF-RRI_supine_, g = 0.44; I^2^ = 0; LF/HF-RRI_tilt_, g = 0.86; I^2^ = 0; LF/HF_tilt_, g = 0.40; I^2^ = 0). As a group, MG patients have altered cardiac autonomic function, including decreased parasympathetic function, lower baroreflex sensitivity, and higher sympathovagal balance at rest and during orthostatic challenges.

## 1. Introduction

Myasthenia gravis (MG) is a rare, chronic autoimmune disease affecting more than 700,000 people worldwide [1]. MG is the largest group of neuromuscular disorders caused by antibodies that target the neuromuscular junction (NMJ), mainly the acetylcholine receptor (AChR), muscle-specific kinase (MuSK), and lipoprotein-associated protein (LRP4). Approximately 80% of patients with ocular symptoms will develop generalized myasthenia within the first 1–2 years after symptom onset. Muscle weakness and fatigability are predominant manifestations of MG [2,3]. However, the disease is also characterized by autonomic dysfunction (AD), which affects the cardiovascular system under the sympathetic and parasympathetic control of the autonomic nervous system [4].

The concept of cardiac involvement in MG was presented for the first time in 1901 when Bramwell described a 30-year-old male myasthenic patient who had tachycardia and heart failure, and defined it as cardiomyasthenia [5]. Since then, research has described myositis and/or myocarditis, conduction and diastolic left ventricle abnormalities, and cardiac autonomic dysfunction (CAD) in MG [6,7,8,9,10,11,12,13,14,15,16,17]. CAD has remained poorly characterized, probably due to subclinical forms and the lack of routine cardiac autonomic evaluation in MG patients. 

Several forms of CAD have been documented among MG patients, comprising altered heart rate variability (HRV), abnormal baroreflex sensitivity (BRS), cardiac arrhythmias, and orthostatic intolerance symptoms [8,11,18,19]. The evidence has not favored one particular type of autonomic dysfunction. Studies into CAD in MG patients have shown both normal and abnormal sympathetic and parasympathetic function, either at rest or in response to hemodynamic challenges [8,9,10,11,12,13,14,15,16]. Some studies suggest that MG patients exhibit sympathetic hyperresponsiveness, with an increased heart rate and elevated blood pressure [10,11,12]. Inconsistency can be explained by a wide spectrum of MG patients with varying disease severity, presence of thymoma, antibody status (anti-AChR antibodies or MuSK antibodies), and variation in autonomic assessment methods.

A recent international expert consensus document recommended that assessment of CAD should include tests of cardiac sympathetic (adrenergic) and cardiovagal function, and quantify its severity. The cardiovagal function is typically assessed through heart rate variation to deep breathing (DBT) and to the Valsalva maneuver (VM), whilst the sympathetic function is evaluated by blood pressure measurements [20]. Other methods such as analysis of HRV in both the time and frequency domain, blood pressure variability (BPV), and BRS measurement have also been used in clinical trials. Furthermore, a combination of autonomic tests should be considered to achieve an accurate measure of total autonomic function [20,21].

The aim of this systematic review with meta-analysis was to determine if there are any differences in cardiovascular autonomic parameters between patients with MG and healthy controls (HCs).

## 2. Methods

This systematic review and meta-analysis was conducted in accordance with Preferred Reporting Items for Systematic Reviews and Meta-Analyses (PRISMA) statement [22] and Meta-analysis of Observational Studies in Epidemiology (MOOSE) guidelines [23] for observational studies. An a priori established but unpublished protocol was followed (available upon request; https://doi.org/10.17605/OSF.IO/ZXCNJ accessed on 3 September 2023).

### 2.1. Information Sources and Search Strategies

Two reviewers (MZK and ŁR) systematically and independently performed a literature screening using PubMed, Web of Science, EMBASE, and SCOPUS, from database inception to 7 July 2023 for studies investigating cardiovascular autonomic parameters in MG. Any disagreements that occurred during full-text screening were resolved via consensus with a third reviewer (NV). There were no restrictions on publication date, age, or setting. Only articles published in English were considered.

The research question was formulated in the PICO (S) format as follows: 

(Participants) People affected by MG diagnosed using standardized criteria; (Intervention) none; (Comparison) healthy controls; (Outcome) cardiovascular autonomic parameters using validated diagnostic tools for autonomic assessment/testing; (S) Observational (case–control, cross-sectional, cohort studies).

We built a comprehensive strategy integrating search terms derived from the PICO elements. The search strategy developed for PubMed was as follows:

(“Myasthenia gravis” OR MuSK MG ) AND (“Autonomic Nervous System Diseases” [mh] OR ANS Disease OR Autonomic Disease OR Autonomic Nervous System OR Autonomic Dysfunction OR parasympathethic OR sympathethic OR Hyperreflexia OR Dysreflexia OR Autonomic OR Dysautonomia OR Orthostatic Intolerance OR Orthostatic Hypotension OR Postural Orthostatic Tachycardia Syndrome OR Postural Tachycardia Syndrome OR Tilt-Table Test OR Valsalva Maneuver OR Baroreflex OR Baroreceptor Reflex OR Blood Pressure OR Breath* Test OR Heart Rate OR Heartbeat OR Cardiac Rate OR Pulse OR Rate Control OR handgrip strength OR Grip Strength OR Hand Strength). The search was then adapted to other databases. 

### 2.2. Eligibility Criteria

The inclusion criteria included the following: (1) Adult (≥18 years) patients diagnosed with MG according to standardized criteria (e.g., diagnosis confirmed by fluctuating ocular and/or extraocular muscle weakness, and fulfillment of at least one of the subsequent criteria: presence of positive AChR or MUSK autoantibodies, electrophysiological results such as repetitive nerve stimulation and/or single-fiber electromyography, observed clinical responsiveness to cholinesterase inhibitors; (2) using validated tools for the detection of CAD, measured by at least one cardiovascular autonomic parameter (e.g., battery of autonomic functions tests: (i) tilt test (blood pressure and/or heart rate variation, (ii) Valsalva maneuver, (iii) handgrip test, (iv) heart rate response to deep breathing, and other non-invasive approaches currently available to evaluate cardiovascular autonomic function such HRV, BRS, and blood pressure variability (BPV)); and (3) [20] observational studies (case–control, cross-sectional, and prospective). 

Studies were excluded if (1) they included pediatric participants; (2) they did not include humans; (3) a control group of healthy controls was not included; or (4) they did not clearly report data regarding autonomic function test in MG patients and/or controls. 

### 2.3. Study Selection 

We adhered to the guidelines outlined in the Cochrane Handbook for Systematic Reviews to select studies for inclusion. During the selection process, through abstract screening, two reviewers (MZK, ŁR) independently screened studies identified through the electronic search engines based on titles and/or abstracts. A third independent reviewer (NV) checked the extracted data. 

The process of study selection involved an initial screening based on titles and/or abstracts, followed by a secondary selection retrieved from this preliminary stage using full-text manuscripts. Relevant items were extracted from the retrieved full-text articles.

Data were stored for identification of the manuscript (e.g., first author’s last name, year of publication, country) and characteristics of participants (e.g., sample size, age, and gender distribution), a diagnostic tool for autonomic assessment (e.g., tilt test, Valsalva maneuver, etc.), and cardiovascular autonomic parameters, as well as data on baseline clinical MG characteristics (disease severity, disease duration, antibody status, presence of thymoma). 

### 2.4. Outcomes

The primary outcomes were expressed as the mean values and the corresponding standard deviations (SDs) of the verified tools of cardiovascular autonomic testing, comparing the values of MG patients with HCs. If the data were presented as medians (interquartile ranges), they were transformed into mean and SDs. Selected parameters are described in Supplementary File S1. We studied baroreflex sensitivity (BRS), Valsalva ratio, E/I ratio, rMSSD, pNN50, and HF indices denoting the parasympathetic ANS, and PSD, SDNN, SDNN index, SDANN, and mean RRI representing the overall HRV modulation. LF indexes mainly reflect the sympathetic cardiac modulation but also reflect parasympathetic (vagal) nervous system activity. Valsalva ratio and E/I ratio represent parasympathetic (cardiovagal) function. BRS is an index of both arms of the ANS, given its involvement in both sympathetic input and vagal output.

### 2.5. Assessment of Risk of Bias 

Studies underwent a methodological quality evaluation for risk of bias using the Newcastle Ottawa Scale (NOS). The NOS scale determinates a maximum of nine points, considering three aspects (subject selection, comparability, and outcome), distributed as follows: 1–3 for low, 4–6 for moderate, and 7–9 for high quality [24].

### 2.6. Data Synthesis

Analyses were performed using PS IMAGO ver. 9.0. Outcomes evaluated by at least two studies (n ≥ 2) were included in the meta-analysis. The main analysis compared cardiovascular autonomic parameters in MG patients and HCs. We calculated the difference between the means of the MG and HC groups using standardized mean differences (SMDs) with 95% confidence intervals (CIs), employing a random-effects model [25]. 

Heterogeneity between the studies was conducted using both the I^2^ metric and χ^2^ statistics. If significant heterogeneity (I^2^ value ≥ 50%, *p* < 0.05) was observed and when there were at least ten studies (N ≥ 10), for a given outcome, we performed a meta-regression analysis using moderators.

Publication bias was evaluated by visual inspection funnel plots, as well as using the objective tests (Begg–Mazumdar Kendall tau, and Egger bias test) [26,27]. To address any potential bias, the trim-and-fill method was employed. If asymmetries were detected, adjustment was made for potential effects of unpublished (trimmed) studies [28].

Significance for all analyses was determined by a *p*-value of less than 0.05.

## 3. Results

**Figure 1:**
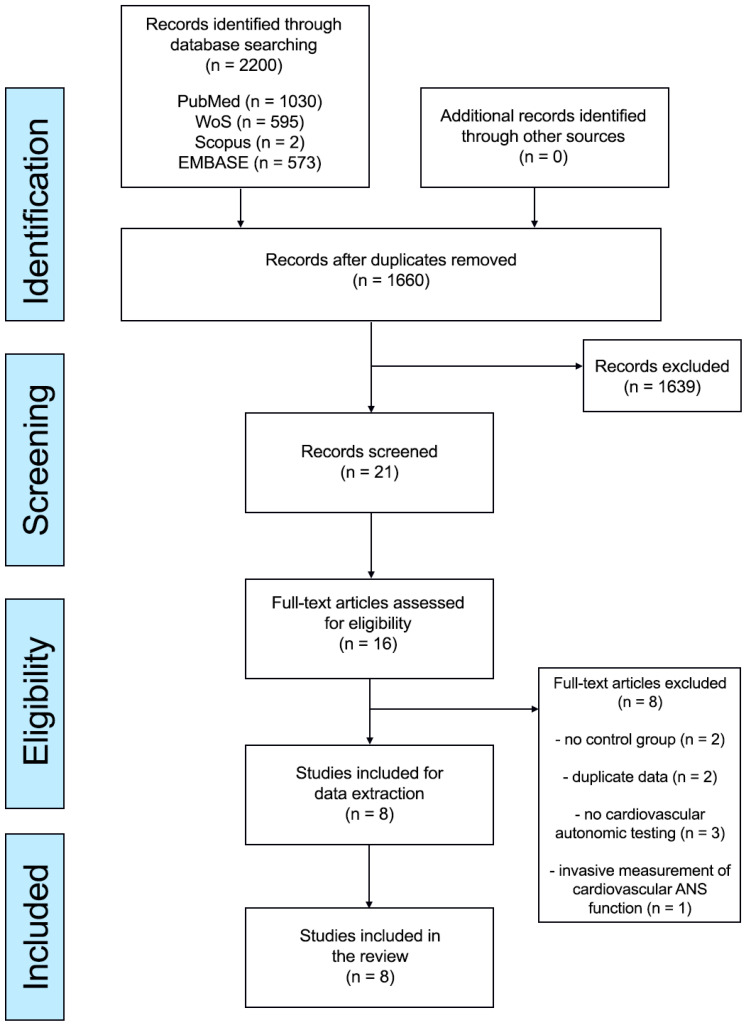
PRISMA flow diagram.

Our search identified 2200 results from 4 electronic databases: PubMed: 1030; Web of Sciences: 595; Scopus: 2; and Embase: 573. After eliminating duplicates, 1660 abstracts were reviewed. From these, 16 full-text articles were considered for eligibility, and eight studies met the inclusion criteria for qualitative and quantitative synthesis. The PRISMA flowchart is shown in Figure 1.

### 3.1. Characteristics of Included Studies and Participant Details

Study and subject characteristics are summarized in Table 1. All the included studies were conducted retrospectively. Six studies were carried out in Europe and two in India. Most of the studies were published during the last ten years. We were able to identify studies reporting at least one abnormal test and seven studies reporting a minimum of two (Table 2). The eight meta-analyzed studies included a total of 755 participants, including 301 subjects with MG and 454 HCs. The participants with MG had a mean age of 45.2 (±13.9) years. A total of 61.9% of the MG patients were women, 64.4% had serum antibodies to the acetylcholine receptor (Abs AchR+), and 23.9% had a thymoma.

All studies included medicated MG subjects. In the HC group, the mean age was 43.9 (±13.2) years, with a share of 60.7% females across eight studies. Seven studies eliminated individuals with additional cardiovascular conditions or pathologies that could potentially involve dysautonomia, such as diabetes. Table 1 summarizes the details of the included studies and participants.

The two domains of analysis (time and frequency) were used in three of the eight studies [8,9,13]. An additional study reported only time domain analyses. 

With respect to cardiovascular reflex tests, six studies [8,9,10,12,13,14] reported sympathetic autonomic function by assessing blood pressure response to a sustained handgrip test, while seven assessed it during an orthostatic challenge. Studies expressed their findings either as a percentage of abnormal results or as absolute values. For the handgrip test, one study used both heart rate (HR) and diastolic blood pressure (dBP), one used mean arterial pressure, and one reported only dBP. Five studies assessed parasympathetic function expressed as HRV in response to standing up and DBT, while six studies reported it using the Valsalva maneuver. Overall, two studies used mean RRI, two studies used RRIV, and one study did not present detailed data. Four studies used BRS at rest, and one during tilt. 

The mean NOS score among the studies was 6 (ranging from 5 to 7). The summarized scores are displayed in Table 1.

### 3.2. Meta-Analysis Results

Comprehensive details of all meta-analysis results, including heterogeneity and publication bias, are provided in Table 3. 

#### 3.2.1. Time Domain HRV Parameters: SDNN; SDANN, rMSSD, pNN50, SDNN Index

In total, five parameters of HRV were analyzed, with the rMSSD and SDNN being the most common.

Data pooled from two studies [8,9] including 96 subjects with MG and 76 HCs demonstrated that patients with MG had a lower pNN50 compared to HCs (g = −1.50, 95% CI −2.52–−0.483 *p* = 0.004). Significant heterogeneity was observed (I^2^ = 82.8, *p* = 0.02).

Data from three studies [8,9,13] including 126 subjects with MG and 106 HCs revealed that MG patients had a lower rMSSD compared to HCs (g = −1.94, 95% CI −3.57, −0.32, *p* = 0.019). There was significant heterogeneity (I^2^ = 95.1, *p* = 0.02) but no evidence of publication bias (Egger = 1.35, *p* = 0.531). (Supplementary File S2).

Data pooled from two studies [8,9] including 96 subjects with MG and 76 HCs demonstrated that MG patients had a lower SDNN index compared to HCs (g = −0.83, 95% CI −1.37–−0.28 *p* = 0.003), with non-significant heterogeneity (I^2^ = 55.5, *p* = 0.13).

There were no differences between MG patients and HCs when comparing SDNN (g = −1.20, 95% CI −3.13–0.73, *p* = 0.22) and SDANN (g = 0.19, 95% CI −0.26–0.64, *p* = 0.51) (Table 3). 

#### 3.2.2. Frequency Domain HRV Parameters at Rest: LFnu, HFnu, LF, HF, LF/HF, LF/HF of HRV, PSD 

The frequency domain HRV measures were used in five studies [8,9,10,11,12,13]. In total, seven parameters were analyzed, with LFnu_supine_ and HFnu_supine_ being the most common.

Four studies [8,9,10,11] conducted LF/HF-RRI_supine_ assessment in 164 subjects with MG and 136 HCs. The pooled data demonstrated that MG patients had a higher LF/HF-RRI_supine_ at rest compared to HCs (g = 0.44, 95% CI 0.21–0.68, *p* < 0.001). There was no heterogeneity (I^2^ = 0; *p* = 0.78) and no evidence of publication bias (Egger = 0.42, *p* = 0.53).

There were no differences between MG patients and HCs at rest when comparing LFnu-RRI_supine_ (g = 1.34, 95% CI −0.88–3.55, *p* = 0.24), LF-RRI_supine_ (g = −0.39, 95% CI −0.39–1.17, *p* = 0.33), HFnu-RRI_supine_ (g = −2.81, 95% CI −7.72–2.10, *p* = 0.26), HF-RRI_supine_ (g = −0.40, 95% CI −1.16–0.36, *p* = 0.30), LF/HF_supine_ (g = 1.80, 95% CI −1.04–4.65, *p* = 0.21), and PSD-RRI_supine_ (g = −0.11, 95% CI −0.46–0.24, *p* = 0.55) (Table 3).

#### 3.2.3. Frequency Domain HRV Parameters in Response to Tilt: LF_tilt_, HF_tilt_, LF/HF_tilt_, LF/HF_tilt_ of HRV, PSD_tilt_

Data pooled from two studies [10,11] including 68 subjects with MG and 60 HCs demonstrated that patients with MG had a lower HF-RRI during tilt (g = −0.75, 95% CI −0.11–−0.39, *p* < 0.001). There was no heterogeneity (I^2^ = 0; *p* = 0.98).

Data pooled from two studies [10,11] including 68 subjects with MG and 60 HCs demonstrated that patients with MG had a higher LF/HF-RRI_tilt_ (g = 0.86, 95% CI 0.50–1.25 *p* < 0.001) and LF/HF_tilt_ compared to HCs (g = 0.81, 95% CI 0.45–1.18 *p* < 0.001), with no heterogeneity (I^2^ = 0, *p* = 0.52, I^2^ = 0, *p* = 0.80, respectively).

There were no differences between MG patients and HCs at rest when comparing LF-RRI_tilt_ (g = −0.13, 95% CI −1.05–1.32, *p* = 0.83) and PSD-RRI_tilt_ (g = −0.24, 95% CI −0.65–0.17, *p* = 0.526) (Table 3).

### 3.3. Baroreflex Sensitivity (BRS)

Data from four studies [8,9,10,11] including 136 subjects with MG and 164 HCs measured BRS. There was a decreased BRS response among patients with MG compared to HCs (g = −0.56, 95% CI −0.89–−0.33, *p* < 0.00) with no heterogeneity (I^2^ = 0.3, *p* = 0.49)

### 3.4. Valsalva Ratio

Data from three studies [12,13,15] including 120 people with MG and 301 HCs measured heart rate response to the Valsalva maneuver (ratio). The Valsalva ratio was used to assess cardiovascular parasympathetic function. There was no decreased Valsalva ratio among people with MG compared to HCs (g= −0.22, 95% CI −0.69–0.25, *p* = 0.36).

### 3.5. Mean R-R Interval

Data from two studies [8,9] including 96 subjects with MG and 76 HCs measured RRI. There was no increased RRI among patients with MG compared to HCs (g = 0.16, 95% CI −0.14–0.47, *p* = 0.29) and no heterogeneity (I^2^ = 81.1, *p* = 0.49)

### 3.6. E/I Ratio

Data from two studies [11,13] including 68 people with MG and 60 HCs measured heart rate response to DBT (E/I ratio). E/I ratio was also used to assess cardiovascular parasympathetic function. There was a decreased heart rate response among people with MG compared to HCs (g = −0.45, 95% CI −1.18–0.24, *p* = 0.01), with significant heterogeneity (I^2^ = 74.7, *p* = 0.047).

### 3.7. Heart Rate and Blood Pressure Parameters at Rest and in Response to Tilt

Five studies [8,10,11,12,14] (patients n = 221, controls n = 373) reported HR at rest (HR_rest_) and four reported HR in response to HUTT (HR_tilt_) (patients n = 146, controls n = 318). There were no differences between MG patients and HCs at rest when comparing HR_supine_ (g = 0.40, 95% CI −0.47–1.26, *p* = 0.37) and HR_tilt_ (g = 0.19, 95% CI −0.57–0.95, *p* = 0.63) (Table 3). Both sBP_supine_ and dBP_supine_ at rest were reported by four studies, [8,10,11,12] and four studies [10,11,12,14], respectively, during HUTT. Similarly, two studies [11,14] reported mBP at rest (mBP_rest_) and in response to HUTT (mBP_tilt_). 

Data pooled from four studies [8,10,11,12] including 204 subjects with MG and 356 HCs demonstrated that MG patients had a higher sBP_supine_ at rest compared to HCs (g = 0.39, 95% CI 0.09–0.68, *p* = 0.01). There was heterogeneity (I^2^ = 84.6; *p* = 0.78) and no evidence of publication bias (Egger = 1.24 *p* = 0.15). 

There were no differences between MG patients and HCs at rest when comparing dBP_supine_ (g = −0.08, 95% CI −0.42–0.27, *p* = 0.66), mBP_supine_ (g = −0.25, 95% CI −0.14–0.66, *p* = 0.20), and during tilt sBP_tilt_ (g = 0.34, 95% CI −0.27–0.94, *p* = 0.28), dBP_tilt_ (g = 0.04, 95% CI −0.61–0.69, *p* = 0.90), mBP_tilt_ (g = −0.04, 95% CI −0.92–0.83, *p* = 0.92). (Supplementary File S2).

## 4. Discussion

Across the eight included studies (including 301 MG patients and 454 HCs), we found evidence that MG patients, compared to HCs, have altered cardiac autonomic functions, including decreased parasympathetic activity, lower baroreflex sensitivity, and higher sympathovagal balance at rest and during orthostatic challenge. Patients with MG had significantly lower E/I ratio, as well as lower pNN50, rMSSD, SDNN index, and HF-RRI_tilt_, and higher systolic blood pressure, LF/HF-RRI_supine_ at rest, and during tilt (LF/HF-RRI_tilt_, LF/HF_tilt_), as measured by HRV, compared to controls. 

The pathophysiological mechanisms through which decreased HRV increases the risk of cardiovascular events and development of CAD are multifactorial. Autoimmunity with immune activation and chronic inflammation is probably a main inducer [29,30]. Previous studies have demonstrated that systemic inflammation markers expressed abnormally in MG patients. In this context, interplay between chronic systemic inflammation and sympathetic nervous system (SNS) overactivation may play a significant role in the autoimmune dysfunction [29]. Inflammation may lead to heightened excitability of premotor sympathetic neurons and suppression of cardiac vagal preganglionic neurons within the medulla [31]. This could lead to an increase in sympathetic outflow to the heart and vasculature, as well as a decrease in parasympathetic outflow to the heart [31]. The result could be elevated blood pressure and a heightened cardiovascular risk [32], as illustrated in our meta-analysis. Another possible mechanism behind AD in MG is cross-reactivity for the skeletal nAChR antibodies, with some idiotypes also binding to ganglionic AChR. Such cross-reactivity has been reported in patients with MG and autoimmune autonomic ganglionopathy (AAG) [33]. In the peripheral nervous system, nicotinic acetylcholine receptors (nAChRs) mediate fast synaptic transmission at autonomic ganglionic (sympathetic, parasympathetic, enteric) and neuromuscular synapses. Furthermore, autoantibodies targeting nAChR in autonomic ganglia have been implicated in AD [34]. Another mechanism of cardiac impairment involving autoantibodies against heart muscle, such as the Ryanodine receptor, adrenergic receptors (beta_1_- and beta_2_), titin, and Kv1.4, may also be present. Myositis and/or myocarditis are the most serious manifestations of MG and are related to poor outcomes [16].

There is evidence of autonomic involvement in individuals with MG [6,15] before and after pharmacological treatment. Cholinergic stimulation induced by AChE inhibitor treatment influences autonomic function, including cardiac function, and improves BRS and HRV in both humans and animal disease models [35,36]. AChE inhibitors also increase the HF while decreasing the LF and sympathovagal balance. Moreover, immunosuppressive agents could potentially affect autonomic function [37], thereby increasing the potential risk of arrhythmia, cardiac hypertrophy, and abnormal vascular remodeling [38]. Corticosteroids hold the potential to impact cardiovascular autonomic function, resulting in improved responses in terms of sympathovagal balance (Prednisone/Prednisolone), as observed in patients with Duchenne Muscular Dystrophy [39]. One study, using echocardiography techniques including Tissue Doppler imaging, found that before pyridostigmine intake, compared to controls, MG patients exhibited lower early diastolic AV-plane velocity and diminished peak systolic strain.

However, differences between the groups were equalized following the administration of the drug, suggesting that pyridostigmine restores diastolic function [7]. Nalbantoglu et al. [15], in a study of 22 MG patients, found no significant difference in parasympathetic tests (RR interval variability and Valsalva ratio) before and one hour after the administration of the drug pyridostigmine. Additionally, lower sympathetic skin response (SSR) amplitudes were observed after drug intake, suggesting that pyridostigmine exerts a noncumulative peripheral sympathetic cholinergic effect. However, the utilization of SSR amplitudes and latencies as a quantitative measure is regarded as debatable [40,41]. Evaluating sudomotor function through the qualitative analysis of SSR based on its presence/absence could potentially be a more reliable method [42].

Activation of the SNS and autonomic imbalance are the first reactions to alterations in cardiac loading or myocardial injury and a common pathway to increased morbidity and mortality [43]. AD may be detected before the onset of hypertension [44], as well as diabetes [45], which are non-autoimmune concomitant comorbidities associated with MG [46,47]. Emerging data suggest that reduced HRV and BRS, representing lower vagal activity, are independently associated with a poor cardiovascular prognosis [48]. In addition, increased sympathetic activity has been considered a plausible cause for both atrial and ventricular arrhythmias, hypertension, and, later, heart failure, all of which are conditions observed in MG patients [46].

In support, a large German study with 1660 participants showed that three-quarters of MG patients reported at least one concurrent disease with cardiovascular diseases prevailing (37%) [47]. The burden of MG, including cardiovascular disease, might be exacerbated by the adverse effects of prolonged corticosteroid use and other immunosuppressive therapies [49,50]. A case-control study with a Dutch population (198 patients) demonstrated a higher prevalence of hypertension (35%) and heart diseases (18%), including heart failure and arrhythmia. They reported a higher prevalence of hypertension and type 2 diabetes than in the general population [50]. Similarly, Harris et al. [46] studied the clinical burden in 1149 MG patients and found that patients with refractory MG had a higher occurrence of hypertension, diabetes, and congestive heart failure in comparison to healthy controls. This finding was also related to prolonged corticosteroid use. Results of a retrospective study in Taiwan revealed that individuals with MG exhibited higher prevalence of hypertension, coronary artery disease, diabetes mellitus, dyslipidemia, and thyroid and cerebrovascular diseases. These comorbidities could potentially be linked to the prolonged use of immunosuppressive treatments for MG [51]. High-quality studies designed to examine the relationship between immunosuppressive/glucocorticoid treatments (such as azathioprine) and ischemic stroke are lacking [52].

Despite the advancements in therapeutic management, MG is still associated with an increased mortality rate, with respiratory causes being the most common [6,16]. A study of 1121 Swedish MG patients found no significant difference in cardiovascular diseases, including heart and cerebrovascular diseases, as causes of death compared to the general population [53].

The results of our meta-analysis suggest that sympathetic function, measured as HRV indices, appears to be relatively normal or less compromised in patients with MG. However, vagal impairment can lead to a relative predominance of sympathetic activity at rest and during cardiovascular challenges.

Previous studies evaluating cardiovascular adrenergic function in patients with MG using different methodological approaches have reported mixed findings, including physiologically preserved, depressed [12], or increased sympathetic reactivity [10,11]. Shukla et al. indicated that MG patients, compared to HCs, showed sympathetic hyperreactivity in terms of HR and BP during HUTT, while showing a lesser increase in cardiovascular parameters during the handgrip test.

The authors state that the smaller increase in HR and BP observed during the handgrip test in MG patients may be attributed to MG-related weakness [12]. In contrast, others found similar cardiovascular responses during the handgrip test or orthostatic challenge [9,10]. Nikolic et al. found a higher percentage of sympathetic dysfunction in thymoma-associated MG patients (80% vs. 34.8%), but similar percentages of abnormal results (handgrip test and orthostatic hypotension test) in AChR-positive MG patients without thymoma and MuSK-positive MG patients compared to controls, respectively [8].

Autonomic measures representing parasympathetic activity, including pNN50, rMSSD, and E/I ratio, appear to be lower for patients with MG than for controls. However, these measures were affected by significant heterogeneity, likely due to the limited number of included studies., differences in in other variables such as the percentage of patients with thymoma, and disease severity. Four studies evaluated BRS, which is widely used to quantify the vagal part of the reflex. These studies had low heterogeneity, and the reliability of the results is high.

All studies included were of medium quality (NOS score, mean 6) and had relatively consistent diagnostic criteria, which allowed for better control of bias. However, one study included patients with concomitant comorbidities (23%), which might interfere with the results [12].

## 5. Limitations

The limitations of the MG studies include their relatively small sample sizes, retrospective design, and the absence of therapy-naïve patients. Furthermore, the quality of the included studies was moderate, and there was a risk of patient selection bias.

Nevertheless, our findings establish a solid foundation for further investigations, also including measures other than cardiac autonomic assessments when examining dysautonomia in MG. Future studies should compare the differences in autonomic function between MG patients with different antibody statuses.

## 6. Conclusions

As a group, MG patients have altered cardiac autonomic function, including decreased parasympathetic function, lower baroreflex sensitivity, and higher sympathovagal balance at rest and during orthostatic challenges.

## Figures and Tables

**Table 1:** Baseline characteristics of included studies.

Study	Country	Total Population (MG, HC)	Mean Age of Population (SD)	Mean Age of MG (SD)	Mean Age of HC (SD)	Total % Female (MG, HC)	Disease Duration (SD)	Disease Severity, n	% Abs AchR+	% Thymoma	NOS
Nalbantoglu et al., 2021 [15]	Turkey	59 (29, 30)	45.95 (14.85)	47.86 (15.08)	44.1 (14.64)	57.58 (55.2; 60)	NR	NR	51.7	10.34	6
Kocabas et al., 2018 [10]	Turkey	60 (30, 30)	44.8 (11.92)	45.9 (12.08)	43.77 (11.1)	48.45 (50; 46.7)	8.6 (6.3)	Remission: n = 12 MGFA I: n = 4 MGFA IIa: n = 9 MGFA IIb: n = 2 MGFA IIIb: n = 0 MGFA IIIa: 2 MGFA IVa: n = 1	73,9	26.66	7
Shukla et al., 2013 [12]	India	302 (61, 241)	NR	NR	NR	NR	NR	NR	NR	6	5
Elsais, 2022 [14]	Norway	34 (17, 17)	45 (13.8)	45 (14)	45 (14.0)	58.8 (58.8; 58.8)	13 (11)	Remission: n = 10 MGFA I: n = 7 MGFA IIa: n = 0 MGFA IIb: n = 0 MGFA IIIb: n = 0 MGFA IIIa: n = 0 MGFA IVa: n = 0	NR	11.76	6
Puneeth et al., 2013 [13]	India	60 (30, 30)	36.05 (13.3)	36.2 (13.6)	35.9 (13.3)	60 (60; 60)	NR	Remission: n = 4 Osserman I: n = 2 Osserman IIa, n = 24	66.7	6.66	6
Peric et al., 2011 [9]	Serbia	42 (21, 21)	52.9 (10.8)	53.2 (9.9)	52.7 (11.9)	47.6 (47.6; 47.6)	7,4 (6)	Remission: n = 7 MGFA I: n =1 MGFA IIa: n = 2 MGFA IIb: n = 9 MGFA IIIa: n = 1 MGFA IIIb: n = 1 MGFA IVa: n = 0	NR	100	6
Nicolic et al., 2014 [8]	Serbia	130 (75, 55)	46.80 (13.88)	46.57 (14.42)	47.12 (13.25)	75.1 (75.3; 74.9)	6.65 (6.32)	Remission: n = 13 MGFA I: n = 24 MGFA IIa: n = 0 MGFA IIb: n = 26 MGFA IIIa: n = 7 MGFA IIIb: n = 5 MGFA IVa: n = 0	69.3	27	6
Zawadka-Kunikowska et al., 2022 [11]	Poland	68 (38, 30)	41.1 (9.9)	42.8 (11.07)	39 (8)	81.75 (86.8; 76.7)	4.4 (2.7)	Remission: n = 0 MGFA I: n = 8 MGFA IIa: n = 19 MGFA IIb: n = 0 MGFA IIIa:11 MGFA IVa: n = 0	60.5	2.63	7

Myasthenia gravis, MG; HC, healthy control group; % positive AChR autoantibodies, %Abs AchR+; Newcastle Ottawa Scale, NOS.

**Table 2:** Summary of included studies.

Study	Diagnostic Tool for Cardiac Autonomic Assessment	Cardiovascular Autonomic Parameters	Main Results
Nalbantoglu et al., 2021 [15]	RRIV Valsalva maneuver	RRIV Valsalva ratio	MG patients (both ocular/generalized) exhibit a subclinical parasympathetic abnormality, which is particularly prominent in the AchR antibody-negative group.
Kocabas et al., 2018 [10]	BRS Head-up tilt test HRV; frequency domain Handgrip test DBT Valsalva maneuver Active standing: HR, sBP, dBP response	Record time: rest, tilt; sBP, dBP, HR; LF, HF, PSD, LF/HF, LF/HF of HRV BRS_rest_, BRS_tilt,_ E/I ratio Valsalva ratio 30:15 ratio HR and BP response to HG Ewing’s battery: percentage of abnormal results	The balance between sympathetic and vagal activity has been disturbed in favor of sympathetic tone and parasympathetic insufficiency has become more prominent.
Shukla et al., 2013 [12]	Head-up tilt: tilt 70° Active standing: 3 min Handgrip test: 3 min Valsalva maneuver R-R interval variation	Record time: rest; RRIV Valsalva ratio HR and sBP, dBP response to HG HR and sBP, dBP response on Valsalva maneuver HR and sBP, dBP response on HUTT HR and sBP, dBP on active standing	Sympathetic hyperreactivity among individuals with MG.
Elsais, 2022 [14]	Head-up tilt Handgrip test	Record time: rest, during, after; HR, mBP	MG patients experiencing fatigue show higher resting heart rates compared to suitably matched HCs. This distinction is more pronounced in patients who are not using acetylcholinesterase inhibitors.
Puneeth et al., 2013 [13]	DBT Handgrip test Valsalva maneuver HRV; frequency and time domains Active standing	Record time: rest; SDNN, rMSSD; LFnu, HFnu, LF/HF E-I difference Valsalva ratio BP response to HG Orthostatic fall in systolic BP	Reduction in the values of HR-based tests, along with a BP-based test (isometric handgrip test), was observed in the study group in comparison to the controls. This reduction indicates a deficiency in parasympathetic activity and a minimal level of sympathetic deficiency.
Peric et al., 2011 [9]	Handgrip test DBT Valsalva maneuver Active standing Orthostatic challenge HRV; frequency and time domains BRS	Record time: rest; LFnu, HFnu, LF/HF of HRV SDNN, SDANN, SDNN index, rMSSD, pNN50 Mean R-R interval BRS_rest_ Ewing’s battery: percentage of abnormal results	Predominantly, there is parasympathetic cardiac impairment in individuals with MG and thymoma.
Nicolic et al., 2014 [8]	Handgrip test Active standing Orthostatic hypotension test Valsalva maneuver BRS HRV; frequency and time domains DBT	Record time: rest HR, sBP, dBP LFnu, HFnu, LF, HF, LF/HF ratio of HRV, PSD of HRV, mean R-R interval SDANN, SDNN, SDNN index, rMSSD, pNN50 Ewing’s battery: percentage of abnormal results	The most prominent autonomic failure was noted among MG patients with thymoma association. Mild parasympathetic abnormalities were observed in AChR-positive thymoma-negative MG patients. MuSK-positive MG patients showed a mild degree of AD.
Zawadka-Kunikowska et al., 2022 [11]	Head-up tilt HRV; frequency domain BPV BRS DBT	Record time: rest, tilt, delta mBP, sBP, dBP, HR LFnu, HFnu, LF, HF, LF/HF, LF/HF of HRV, PSD of HRV LFnu, HFnu, LF, HF, LF/HF, LF/HF of HRV, PSD of sBPV BRS_rest,_ E/I ratio	CAD with predominant parasympathetic dysfunction.

MG, myasthenia gravis, C, control group; R-R interval variation, RRIV; R-R intervals; deep breathing test, DBT, baroreflex sensitivity (BRS); heart rate, HR; systolic blood pressure sBP; diastolic blood pressure dBP; mean blood pressure, mBP; heart rate variability, HRV; blood pressure variability, BPV; low frequency, LF; high frequency, HF; power spectral density, PSD, index of sympathovagal balance LF/HF, expiration/inspiration ratio the longest R-R interval during inspiration E/I ratio; handgrip test, HG; head-up tilt test, HUTT, expiration-inspiration difference, E-I difference; ratio between longest R-R interval at or around the 30th beat and shortest R-R interval at or around the 15th beat 30:15 ratio; SDNN, mean standard deviation of all normal RR intervals, standard deviation of the averages of NN intervals in all 5-min segments, SDANN; mean of the standard deviations of all NN intervals for all 5-min segments, SDNN index, square root of the mean of squared differences between successive beat intervals, rMSSD; number of pairs of adjacent NN intervals differing more by than 50 ms divided by the total number of all NN intervals, pNN50; nu, normalized units.

**Table 3:** Meta-analysis results of cardiac autonomic parameters versus healthy controls.

	Number of Data Sets	Sample MG	Sample C	Effect Size (95%CI)	*p*	I^2^	*p* of I^2^	Publication Bias
HR_supine_	5	221	373	0.40 (−0.47; −1.26)	0.37	94.8	<0.001	N/A
sBP_supine_	4	204	356	0.39 (0.09; −0.68)	0.01	56.1	0.09	No
dBP_supine_	4	204	356	−0.08 (−0.42; −0.27)	0.66	68.1	0.013	No
mBP_supine_	2	55	47	0.25 (−0.14, 0.66)	0.20	0.0	0.412	N/A
LFnu_supine_	4	164	136	1.34 (−0.88, 3.55)	0.24	98.5	<0.001	No
HFnu_supine_	4	164	134	−2.81 (−7.72, 2.10)	0.26	99.7	<0.001	No
LF_supine_	2	68	60	0.39 (−0.39, 1.17)	0.33	79.2	0.028	N/A
HF_supine_	2	68	60	−0.40 (−1.16, 0.36)	0.30	77.9	0.033	N/A
LF/HF_supine_	3	68	60	1.80 (−1.04, 4.65)	0.21	98.4	<0.001	No
LF/HF-RRI_supine_	4	164	136	0.44 (0.21, 068)	**<0.001**	0	0.79	No
RRI_supine_	2	96	76	0.16( −0.14, –0.47	0.66	61.7	0.11	N/A
PSD-RRI_supine_	2	68	60	−0.11 (−0.46, 0.24)	0.55	0	0.47	N/A
SDNN	3	126	106	−1.20 (−3.13, 0.73)	0.22	97.2	<0.001	No
SDANN	2	96	76	0.19 (−0.26, 064)	0.41	44.5	0.179	N/A
rMSSD	3	126	106	−1.94 (−3.57, −0.32)	**0.02**	95.1	<0.001	No
pNN50	2	96	76	−1.2 (−3.134, 0.735)	**0.004**	82.8	0.016	N/A
SDNN index	2	96	76	−0.83 (−1.37, −0.28)	**0.003**	55.5	0.13	N/A
BRS_supine_	4	136	164	−0.56 (−0.80, −0.33)	**<0.001**	0.3	0.49	No
HR_tilt_	4	146	318	0.19 (−0.57–0.95)	0.63	90.5	<0.001	No
sBP_tilt_	3	129	301	0.34 (−0.27–0.94)	0.28	83.8	0.04	No
dBP_tilt_	3	129	301	0.04 (−0.61–0.69)	0.90	85.7	0.004	No
mBP_tilt_	2	55	47	−0.04 (−0.92–0.83)	0.92	77.3	0.036	N/A
LF_tilt_	2	68	60	0.13 (−1.05, 1.32)	0.83	90.9	<0.001	N/A
HF_tilt_	2	68	60	−0.75 (−1.11, −0.39)	**0.00**	0	0.98	N/A
LF/HF-RRI_tilt_	2	68	60	0.86 (0.50, 1.23)	**<0.001**	0.	0.96	N/A
LF/HF_tilt_	2	68	60	0.40 (0.05, 0.75)	**0.02**	0	0.57	N/A
PSD-RRI_tilt_	2	68	60	−0.24 (−0.65, 0.17)	0.26	25.8	0.246	N/A
Valsalva ratio	3	120	301	−0.22 (−0.69–0.25)	0.36	72.2	0.045	No
E/I ratio	2	68	60	−0.45 (−0.80, −0.09)	**0.01**	74.7	0.047	N/A

MG, myasthenia gravis; C, control group; N/A = not applicable (<3 studies publication bias not applicable). Bold values represent significant results (*p*-value < 0.05).

## Data Availability

The datasets generated during and/or analyzed during the current study are available from the corresponding author on reasonable request.

## Supplementary Materials

The following supporting information can be downloaded at: https://www.mdpi.com/article/10.3390/neurolint15030071/s1. Supplementary File S1 (Table S1: Selected parameters), Supplementary File S2 (Figure S1: Baroreflex sensitivity and Figure S2: Low frequency to high frequency ratio of HRV).
