# Supplementary material for: Altered Cardiac Autonomic Regulation in Individuals with Myasthenia Gravis—A Systematic Review and Meta-Analysis
Source: Neurol Int. 2023 Sep 8;15(3):1140–54. doi: 10.3390/neurolint15030071 (PMC10537350; doi:10.3390/neurolint15030071)
Supplement: Supplementary file 1 [file neurolint-15-00071-s001.zip › neurolint-2538452-supplementary.pdf]

Supplementary File S1

Table S1. Selected parameters.

Selected Parameter	Description
Mean RRI	RR intervals in milliseconds, ms
SDNN	Mean standard deviation of all normal RR intervals, ms
RMSSD	Square root of the mean of squared differences between successive beat intervals, ms)
pNN50	percentage of adjacent NN intervals differing by more than 50 ms/ percentage of adjacent NN intervals differing by more than 50 ms ,%),
SDNN index	Mean of the standard deviations of all NN intervals for all 5-min segments, ms
SDANN	Standard deviation of the averages of NN intervals in all 5-min segments, ms
LF	Low frequency, ms^2 and normalized units, nu
HF	High frequency, ms^2 and normalized units, nu
LF/HF	Index of sympathovagal balance
LF/HF-RRI	Low frequency to high frequency ratio of HRV
PSD of HRV.	Power spectral density, ms^2
BRS	Baroreflex sensitivity calculated as the slope of the regression between spontaneous sequences of blood pressure changes and simultaneously occurring variations of RR intervals, ms/mmHg using sequence method
Valsalva ratio	Ratio of the longest R-R interval after the Valsalva maneuver to the shortest interval during the maneuver),
E/I ratio	Expiration/inspiration ratio the longest R-R interval during inspiration),
HR	Heart rate, 1/min
sBP	Systolic blood pressure, mmHg
dBP	Diastolic blood pressure, mmHg
mBP	Mean blood pressure, mmHg

References [8–15] are cited in main text

Supplementary File S2

A

■ Effect size of each study | Confidence interval of effect size
◆ Estimated overall effect size - - Overall effect size value

ID	Hedges'g	Std. Error	Lower	Upper	p–value	Weight	Weight (%)
Kocabas et al. 2018	–0.41	0.26	–0.92	0.10	0.12	14.64	20.78
Peric et al. 2011	–1.02	0.33	–1.67	–0.37	0.00	9.17	13.01
Nikolic et al. 2014	–0.49	0.18	–0.85	–0.14	0.01	30.62	43.47
Zawadka–Kunikowska et al. 2022	–0.58	0.25	–1.07	–0.09	0.02	16.02	22.74
Overall	–0.56	0.12	–0.80	–0.33	0.00		

Model: Random–effects model
Heterogeneity: Tau–squared = 0.00, H–squared = 1.00, I–squared = 0.00
Homogeneity: Q = 2.43, df = 3, p–value = 0.49
Test of overall effect size: z = –4.72, p–value = 0.00

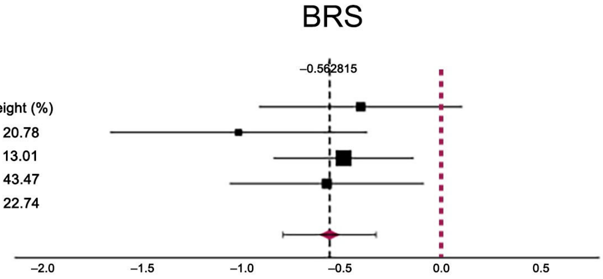

B

ID	Hedges'g	Std. Error	Lower	Upper	p–value	Weight	Weight (%)
Puneeth et al. 2013	–0.85	0.27	–1.38	–0.32	0.00	3.71	48.74
Zawadka–Kunikowska et al. 2022	–0.12	0.24	–0.60	0.36	0.62	3.90	51.26
Overall	–0.47	0.36	–1.18	0.24	0.19		

Model: Random–effects model
Heterogeneity: Tau–squared = 0.20, H–squared = 3.96, I–squared = 0.75
Homogeneity: Q = 3.96, df = 1, p–value = 0.05
Test of overall effect size: z = –1.31, p–value = 0.19

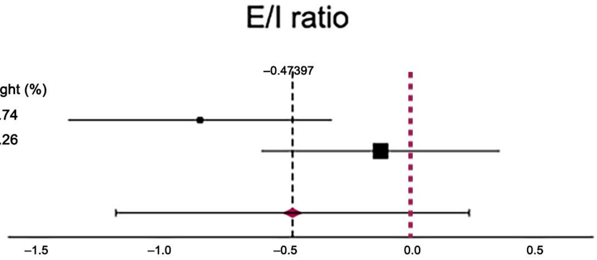

C

ID	Hedges'g	Std. Error	Lower	Upper	p–value	Weight	Weight (%)
Peric et al. 2011	–1.18	0.34	–1.84	–0.52	0.00	4.87	37.77
Nikolic et al. 2014	–0.61	0.18	–0.96	–0.25	0.00	8.03	62.23
Overall	–0.83	0.28	–1.37	–0.28	0.00		

Model: Random–effects model
Heterogeneity: Tau–squared = 0.09, H–squared = 2.25, I–squared = 0.56
Homogeneity: Q = 2.25, df = 1, p–value = 0.13
Test of overall effect size: z = –2.96, p–value = 0.00

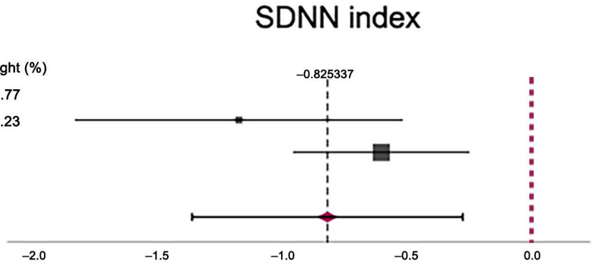

D

ID	Hedges'g	Std. Error	Lower	Upper	p–value	Weight	Weight (%)
Peric et al. 2011	–2.08	0.39	–2.84	–1.31	0.00	1.66	44.67
Nikolic et al. 2014	–1.03	0.19	–1.40	–0.66	0.00	2.06	55.33
Overall	–1.50	0.52	–2.52	–0.48	0.00		

Model: Random–effects model
Heterogeneity: Tau–squared = 0.45, H–squared = 5.80, I–squared = 0.83
Homogeneity: Q = 5.80, df = 1, p–value = 0.02
Test of overall effect size: z = –2.89, p–value = 0.00

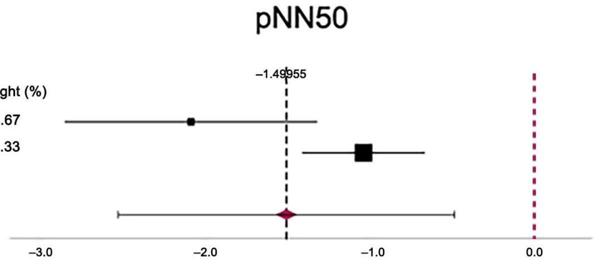

E

ID	Hedges'g	Std. Error	Lower	Upper	p–value	Weight	Weight (%)
Puneeth et al. 2013	–3.47	0.42	–4.29	–2.65	0.00	0.47	32.40
Peric et al. 2011	–1.85	0.37	–2.59	–1.12	0.00	0.48	32.91
Nikolic et al. 2014	–0.61	0.18	–0.96	–0.25	0.00	0.50	34.69
Overall	–1.94	0.83	–3.57	–0.32	0.02		

Model: Random–effects model
Heterogeneity: Tau–squared = 1.96, H–squared = 20.56, I–squared = 0.95
Homogeneity: Q = 43.33, df = 2, p–value = 0.00
Test of overall effect size: z = –2.34, p–value = 0.02

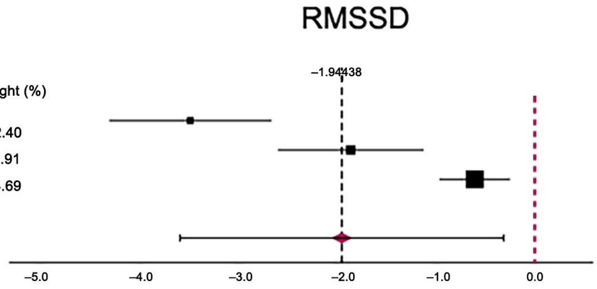

Figure S1. Baroreflex sensitivity, BRS (**A**) [8-11]; expiration/inspiration ratio, E/I (**B**) [11,13]; Mean of the standard deviations of all NN intervals for all 5 min segments, SDNN index (**C**) [8,9]; Number of pairs

of adjacent NN intervals differing more than 50 ms divided by the total number of all NN intervals, pNN50 (**D**) [8,9]; Square root of the mean of squared differences between successive beat intervals, RMSSD (**E**) [8,9,13]. References are cited in main text

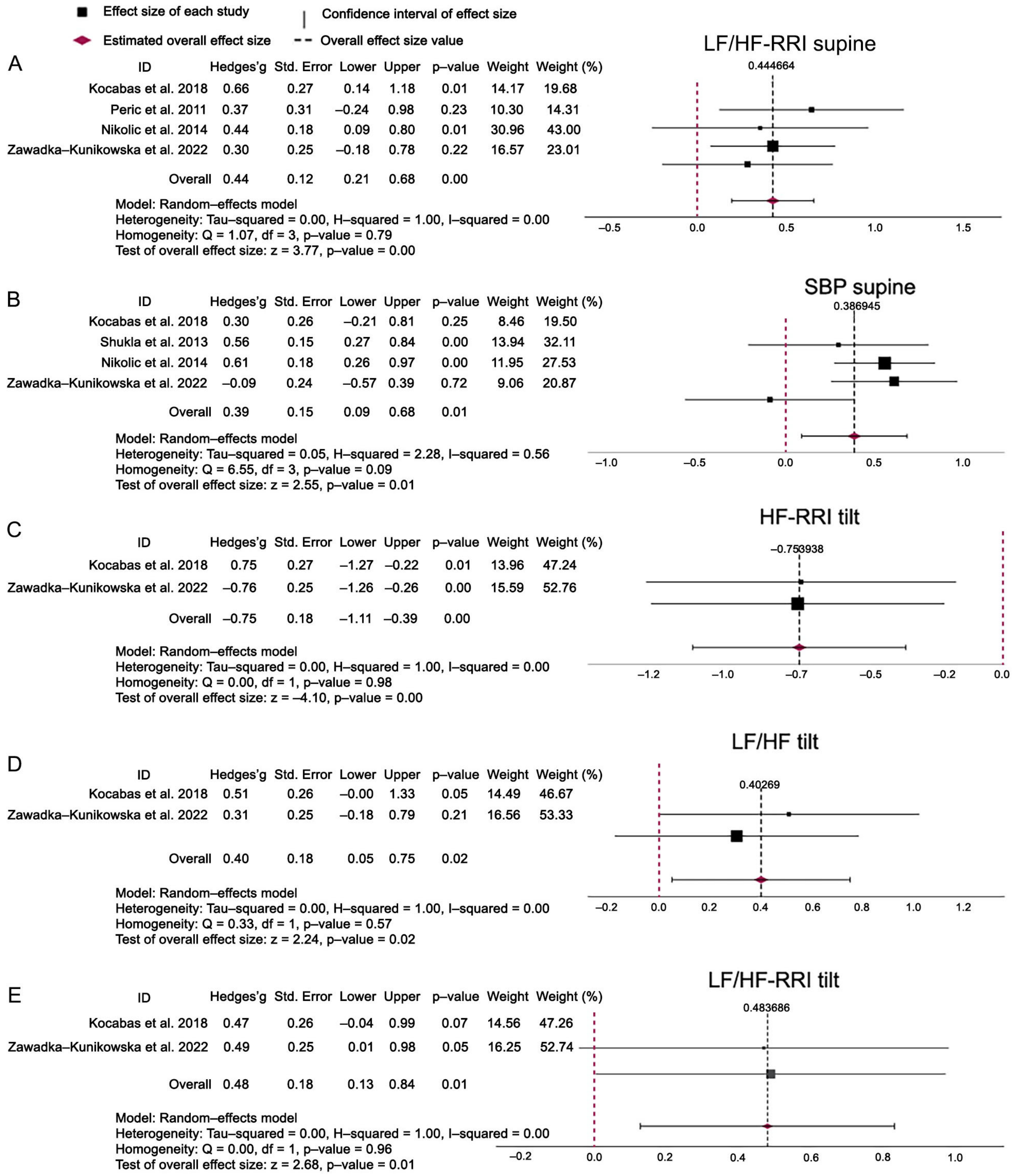

A

ID	Hedges'g	Std. Error	Lower	Upper	p–value	Weight	Weight (%)
Kocabas et al. 2018	0.66	0.27	0.14	1.18	0.01	14.17	19.68
Peric et al. 2011	0.37	0.31	−0.24	0.98	0.23	10.30	14.31
Nikolic et al. 2014	0.44	0.18	0.09	0.80	0.01	30.96	43.00
Zawadka–Kunikowska et al. 2022	0.30	0.25	−0.18	0.78	0.22	16.57	23.01
Overall	0.44	0.12	0.21	0.68	0.00		

Model: Random–effects model
Heterogeneity: Tau–squared = 0.00, H–squared = 1.00, I–squared = 0.00
Homogeneity: Q = 1.07, df = 3, p–value = 0.79
Test of overall effect size: z = 3.77, p–value = 0.00

B

ID	Hedges'g	Std. Error	Lower	Upper	p–value	Weight	Weight (%)
Kocabas et al. 2018	0.30	0.26	−0.21	0.81	0.25	8.46	19.50
Shukla et al. 2013	0.56	0.15	0.27	0.84	0.00	13.94	32.11
Nikolic et al. 2014	0.61	0.18	0.26	0.97	0.00	11.95	27.53
Zawadka–Kunikowska et al. 2022	−0.09	0.24	−0.57	0.39	0.72	9.06	20.87
Overall	0.39	0.15	0.09	0.68	0.01		

Model: Random–effects model
Heterogeneity: Tau–squared = 0.05, H–squared = 2.28, I–squared = 0.56
Homogeneity: Q = 6.55, df = 3, p–value = 0.09
Test of overall effect size: z = 2.55, p–value = 0.01

C

ID	Hedges'g	Std. Error	Lower	Upper	p–value	Weight	Weight (%)
Kocabas et al. 2018	0.75	0.27	−1.27	−0.22	0.01	13.96	47.24
Zawadka–Kunikowska et al. 2022	−0.76	0.25	−1.26	−0.26	0.00	15.59	52.76
Overall	−0.75	0.18	−1.11	−0.39	0.00		

Model: Random–effects model
Heterogeneity: Tau–squared = 0.00, H–squared = 1.00, I–squared = 0.00
Homogeneity: Q = 0.00, df = 1, p–value = 0.98
Test of overall effect size: z = –4.10, p–value = 0.00

D

ID	Hedges'g	Std. Error	Lower	Upper	p–value	Weight	Weight (%)
Kocabas et al. 2018	0.51	0.26	−0.00	1.33	0.05	14.49	46.67
Zawadka–Kunikowska et al. 2022	0.31	0.25	−0.18	0.79	0.21	16.56	53.33
Overall	0.40	0.18	0.05	0.75	0.02		

Model: Random–effects model
Heterogeneity: Tau–squared = 0.00, H–squared = 1.00, I–squared = 0.00
Homogeneity: Q = 0.33, df = 1, p–value = 0.57
Test of overall effect size: z = 2.24, p–value = 0.02

E

ID	Hedges'g	Std. Error	Lower	Upper	p–value	Weight	Weight (%)
Kocabas et al. 2018	0.47	0.26	−0.04	0.99	0.07	14.56	47.26
Zawadka–Kunikowska et al. 2022	0.49	0.25	0.01	0.98	0.05	16.25	52.74
Overall	0.48	0.18	0.13	0.84	0.01		

Model: Random–effects model
Heterogeneity: Tau–squared = 0.00, H–squared = 1.00, I–squared = 0.00
Homogeneity: Q = 0.00, df = 1, p–value = 0.96
Test of overall effect size: z = 2.68, p–value = 0.01

Figure S2. Low frequency to high frequency ratio of HRV, LF/HF-RRI (**A**) [8-11]; Systolic blood pressure, SBP (**B**)[8,10-12]; High frequency of HRV, HF-RRI (**C**) [10,11]; index of sympathovagal balance LF/HF$_{TILT}$ (**D**) [10,11]; Low frequency to high frequency ratio of HRV LF/HF-RRI$_{TILT}$ (**E**) [10,11]. References are cited in main text
